# Implementation of a novel program to support colorectal cancer screening in a community health center consortium before and after the onset of COVID-19: a qualitative study of stakeholders’ perspectives

**DOI:** 10.1186/s43058-023-00439-x

**Published:** 2023-05-22

**Authors:** Eduardo J. Santiago-Rodríguez, Kristin S. Hoeft, Kara Lugtu, Matthew McGowen, David Ofman, Jaime Adler, Ma Somsouk, Michael B. Potter

**Affiliations:** 1 Department of Epidemiology and Biostatistics, University of California, San Francisco, CA USA; 2 Department of Preventive and Restorative Dental Sciences, University of California, San Francisco, CA USA; 3 Department of Family and Community Medicine, University of California, 500 Parnassus Avenue, MU3E – Room 330, San Francisco, CA 94143 USA; 4 San Francisco Community Clinic Consortium, San Francisco, CA USA; 5 AcademyHealth, Washington, D.C. USA; 6 Division of Gastroenterology, Department of Medicine, University of California, San Francisco, CA USA

**Keywords:** Colorectal cancer screening, Community health centers, Qualitative research, Consolidated Framework for Implementation Research, COVID-19, San Francisco

## Abstract

**Background:**

In 2017, the San Francisco Cancer Initiative (SF CAN) established the Colorectal Cancer (CRC) Screening Program to provide technical assistance and financial support to improve CRC screening processes, and outcomes in a consortium of community health centers (CHCs) serving low-income communities in San Francisco. The purpose of this study was twofold: to evaluate the perceived influence of the support provided by the CRC Screening Program’s Task Force on CRC screening processes and outcomes in these settings and to identify facilitators and barriers to SF CAN-supported CRC screening activities before and after the onset of the COVID-19 pandemic.

**Methods:**

Semi-structured key informant interviews were conducted with consortium leaders, medical directors, quality improvement team members, and clinic screening champions. Interviews were audio-recorded, professionally transcribed, and analyzed for themes. The Consolidated Framework for Implementation Research (CFIR) was used to develop the interview questions and organize the analysis.

**Results:**

Twenty-two participants were interviewed. The most commonly cited facilitators of improved screening processes included the expertise, funding, screening resources, regular follow-up, and sustained engagement with clinic leaders provided by the task force. The most salient barriers identified were patient characteristics, such as housing instability; staffing challenges, such as being understaffed and experiencing high staff turnover; and clinic-level challenges, such as lack of ability to implement and sustain formalized patient navigation strategies, and changes in clinic priorities due to the COVID-19 pandemic and other competing health care priorities.

**Conclusions:**

Implementing CRC screening programs in a consortium of CHCs is inherently challenging. Technical assistance from the Task Force was viewed positively and helped to mitigate challenges both before and during the pandemic. Future research should explore opportunities to increase the robustness of technical assistance offered by groups such as SF CAN to support cancer screening activities in CHCs serving low-income communities.

**Supplementary Information:**

The online version contains supplementary material available at 10.1186/s43058-023-00439-x.

Contributions to the literature

- Describes a novel approach for NIH-supported cancer centers to support colorectal cancer (CRC) screening activities in community health centers (CHCs) within their catchment areas.
- Although the CRC Screening Program facilitated CRC screening documentation and processes, barriers commonly encountered in CHCs and the COVID-19 pandemic limited a sustained progress in CRC screening rates.
- The Consolidated Framework for Implementation Research provides a useful structure for evaluating interventions such as SF CAN’s CRC Screening Program but could be improved by expanding the scope of the *outer setting* to accommodate external events that affect the implementation of programs, such as natural disasters and pandemics.

## Background

Cancer is the leading cause of death in San Francisco, CA [1]. The cancer sites with the highest rates of incidence and mortality in the city are the prostate, breast, lung, colon, and rectum [2, 3]. A commonality of these types of cancer is the existence of screening strategies with demonstrated success in reducing cancer mortality [4]. However, cancer screening rates are low in marginalized populations exposed to social risks such as poverty, housing instability, and structural racism [5–7]. In the USA, care for these populations is often provided by federally qualified community health centers, simply referred to as community health centers (CHCs). CHCs deliver care to individuals regardless of their ability to pay, and they are often organized geographically into consortia to support important shared strategic initiatives [8]. The San Francisco Community Clinic Consortium (SFCCC) is one such organization, offering comprehensive primary, preventive, and ambulatory care at 12 CHCs, serving more than 100,000 uninsured, low-income individuals per year [9]. Other services offered at CHCs include mental health, dental and vision care, health promotion, and disease prevention education.

Recognizing the responsibility of NIH-funded Comprehensive Cancer Centers to address cancer disparities within their catchment areas, the University of California San Francisco (UCSF) launched the San Francisco Cancer Initiative (SF CAN) in 2017. SF CAN is a collaborative endeavor between academia, health care systems, government, community groups, and residents of the area, with a primary focus to reduce inequities in access to cancer prevention (i.e., screening) and quality health care [10]. Since 2018, the SF CAN’s Colorectal Cancer (CRC) Screening Program has collaborated with the SFCCC to support CRC screening within its member CHCs. The CRC Screening Program work is led by a Task Force whose members support the SFCCC leadership team’s CRC screening efforts with information about screening tests, trainings for its quality improvement (QI) teams, and guidance on reporting and tracking screening rates. In addition, the CRC Screening Program offers technical assistance and financial support in the form of annual stipends. CHCs are responsible for setting screening goals, identifying activities to work on during the year, assigning a point of contact to lead the work (usually one or two QI staff and a screening champion per CHC), conducting data tracking and reporting, implementing evidence-based interventions, and utilizing QI tools. Task Force members have regular meetings with CHCs’ QI teams to discuss strategies and exchange experiences. In 2020, many of these activities were temporarily halted or changed in response to the COVID-19 pandemic.

The purpose of this study was to evaluate the partnership between SF CAN and the SFCCC to support improved CRC screening processes and outcomes over the first 3 years of collaboration, both before and during the onset of the COVID-19 pandemic. We used the Consolidated Framework for Implementation Research (CFIR) to develop a comprehensive set of domains of inquiry and interviewed stakeholders with diverse roles in the implementation of clinic-level CRC screening activities with support from the CRC Screening Program [11]. Obtaining input from people with distinct perspectives allowed us to identify the key factors that need to be sustained or adjusted across system levels. The timing of this evaluation provided an unusual opportunity to understand barriers and facilitators to CRC screening activities and the support of the CRC Screening Program both before and during a major public health crisis.

## Methods

### Study setting

SFCCC’s 12 CHCs provide care at 27 primary care and wellness sites serving 112,600 individuals, most of whom reside in San Francisco [6]. During the study period, CRC screening was recommended for average-risk adults aged 50–75 [12]. The policy of most SFCCC clinics was to offer CRC screening to this group with annual fecal immunochemical tests (FIT), with colonoscopy provided to those with abnormal FIT or with other personal or family history that places them at elevated risk. At baseline, the primary method of providing FIT was to have them ordered by clinicians during routine office visits, with test completion instructions provided by a medical assistant after the orders were placed.

CHCs willing to work with the Task Force to develop and implement tailored strategies to improve screening rates within their target patient population received up to $5000 per year from SF CAN. CHCs signing up to receive such support were assisted with process mapping their screening workflows, identifying bottlenecks, and identifying evidence-based strategies to improve them. Each participating CHC developed its own set of goals tailored to its needs and was held accountable for addressing these goals through quarterly meetings, site visits by the Task Force, and annual reporting.

### Study design

We used a qualitative approach to evaluate the support provided by the CRC Screening Program to SFCCC’s CHCs before and after the onset of the COVID-19 pandemic. CFIR was used to focus the evaluation, develop the interview guide, and structure the analysis. Briefly, CFIR is a conceptual framework that allows a comprehensive examination of factors that affect the implementation of interventions at multiple levels. Described by Damschroder and colleagues in 2009, CFIR considers 39 elements in five areas or domains [11]. The domains are intervention characteristics, outer setting, inner setting, characteristics of individuals, and process. CFIR has been employed widely in the evaluation of CRC screening programs [13–15].

The interview guide was developed by two researchers external to SF CAN (EJS and KSH) and included questions about interviewees’ roles, CHCs’ goals, support provided by the CRC Screening Program, communication and planning, and implementation of CRC screening activities. At the end of each interview, we included a set of questions about COVID-19 to obtain more details of its impact on CHCs and their needs and recommendations for the future (see Additional file 1).

We used the standards for reporting qualitative research (SRQR) to present the study design and findings (see Additional file 2) [16]. The Institutional Review Board of the University of California, San Francisco approved this study (number: 20–32801).

### Selection of respondents

**Fig. 1:**
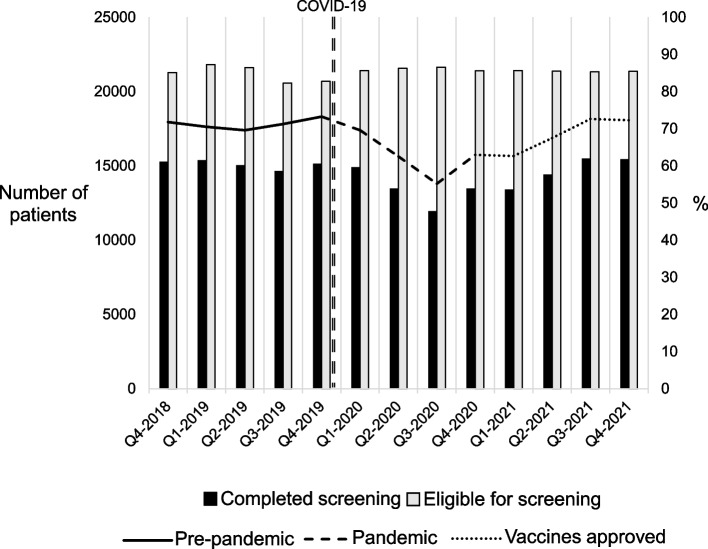
Colorectal cancer screening at participating SFCCC’s CHCs before and after the onset of COVID-19

Prospective interview participants were selected using a purposeful sampling strategy to ensure a diverse representation of (1) the role of people involved in the implementation of CRC screening at CHCs among the informants and (2) CHC characteristics (e.g., clinic size, location, commitment to program) [17]. After identifying the key personnel participating in the CRC Screening Program (i.e., SFCCC leadership, medical directors, QI staff, and screening champions from CHCs; at least one person from each role per CHC), we sent an email describing the purpose of the study and invited them to participate. Approximately 1 week after sending that email, we contacted the potential interviewees, asked about their willingness to participate and scheduled the interviews for those interested. The response rate was 81%. Information about the number of patients eligible for screening and those completing screening at the CHCs participating in the study was provided by the SFCCC and is presented in Fig. 1.

### Data collection

Two days before the scheduled interview, participants received via email an information sheet detailing the purpose of the study and procedures. Consent to participate in the study was obtained verbally at the start of each interview. Interviews lasted 25 to 40 min and were recorded and professionally transcribed. After completing the interview, each participant received a $50 gift card. All interviews were conducted by one of the researchers external to the CRC Screening Program (EJS).

### Data analysis

Transcripts of all interviews were imported and coded using the Dedoose Software (version 9.0.17) [18]. A codebook was developed by two members of the research team (EJS and KSH) using a deductive approach based on CFIR domains, constructs, and subconstructs. Emergent codes were also added after reading the transcripts, mostly for questions about COVID-19, needs, and recommendations. The research team met to discuss the codes and achieve consensus on the list that would be used. After completing the coding, the coded excerpts were compared across informants’ roles, and framework mapping was used to organize the findings.

## Results

Seventeen semi-structured interviews were conducted between February 2021 and March 2021 using the Zoom platform. In five interviews, two members of the clinic with the same or similar roles participated together, for a total of 22 informants. Informants had different roles in implementing CRC screening activities at the CHCs: two SFCCC leaders (chief medical officer and vice president of compliance and quality), medical directors from four CHCs, nine members of QI teams (six managers and three directors), and seven screening champions (six nurses and one medical assistant). Five CHCs that had received at least 1 year of technical assistance and funding were represented. Characteristics of informants and patients receiving services at each CHC are included in Table 1.

**Table 1:** Characteristics of participating community health centers and informants

	All	A	B	C	D	E
**Community health centers, *n* = 5**						
Total of patients, *n* (row %)	72,088	4574 (6.3)	3979 (5.5)	48,987 (68.0)	1276 (1.8)	13,272 (18.4)
Patients 50–75 years, *n* (column %)	20,508 (28.4)	1566 (34.2)	340 (8.5)	15,647 (31.9)	340 (26.6)	2615 (19.7)
Gender, *n* (column %)						
Male	19,506 (27.1)	2715 (59.3)	1916 (48.2)	8839 (18.0)	323 (25.3)	5713 (43.0)
Female	21,326 (29.6)	1310 (28.6)	1721 (43.3)	11,646 (23.8)	338 (26.5)	6311 (47.6)
Trans	214 (0.3)	81 (1.8)	19 (0.5)	7 (0)	0 (0)	107 (0.8)
Others	144 (2.6)	86 (1.9)	308 (7.7)	10 (0)	580 (45.4)	860 (6.5)
Unknown	29,198 (40.5)	382 (0.8)	15 (0.4)	28,485 (58.1)	35 (2.7)	281 (2.1)
Race and ethnicity, *n* (column %)						
Non-Hispanic White	5254 (7.3)	1362 (29.8)	766 (19.3)	2040 (4.2)	139 (10.9)	947 (7.1)
Non-Hispanic Black	2769 (3.8)	958 (20.9)	952 (23.9)	475 (1.0)	146 (11.4)	238 (1.8)
Non-Hispanic Asian/Pacific Islander	46,318 (64.3)	391 (8.5)	1028 (25.8)	44,504 (90.8)	113 (8.9)	282 (2.1)
Non-Hispanic American Indian/Alaska Native	196 (2.7)	106 (2.3)	29 (0.7)	48 (0)	10 (0.8)	3 (0)
Hispanic	12,264 (17.0)	885 (19.3)	218 (5.4)	340 (0.7)	554 (43.4)	10,267 (77.4)
Others	702 (1.0)	361 (7.9)	271 (6.8)	0 (0)	20 (1.6)	50 (0.4)
Unknown	4585 (6.4)	511 (11.1)	715 (18.0)	1580 (3.2)	294 (23.0)	1485 (11.2)
Homeless patients, n (column %)	5762 (8.0)	3279 (71.7)	820 (20.6)	77 (0.2)	935 (73.3)	651 (5.0)
**Informants, *n* = 22**		*n* = 5	*n* = 3	*n* = 3	*n* = 3	*n* = 6
Role						
SFCCC leadership	2	–	–	–	–	–
Medical directors	4	1	1	1	0	1
Quality improvement staff	9	3	1	1	2	2
Screening champions	7	1	1	1	1	3
Years in role, median (min, max)	2.3 (0.5, 15.5)	2.5 (1.3, 6)	1.3 (1, 2)	5.5 (2, 15.5)	2 (1.3, 2)	4 (0.5, 11.5)

Informants described facilitators and barriers related to the implementation of CRC screening activities at the CHCs, which we organized within each of the five CFIR domains. Findings were grouped based on informants’ roles and are summarized below in Tables 2 and 3. Some quotes that best represent the themes are included in the text, and all quotes can be found in Additional file 3: Tables S1 and S2.

**Table 2:** Facilitators to CRC screening activities in SFCCC by informants’ roles according to CFIR

CFIR domains	CFIR constructs and subconstructs	Themes	Roles			
			**SFCCC leadership**	**Medical directors**	**QI staff**	**Screening champions**
Intervention characteristics	Adaptability	Approach	X	X	X	X
	Design quality and packaging	Accountability		X	X	X
		Expertise	X		X	
		Materials		X	X	X
		Trainings				X
	Cost	Stipends	X	X	X	X
Inner setting	Networks and communications	Communication			X	X
		Collaboration within clinics			X	X
	Implementation climate—goals and feedback	Discussing goals and strategies as a team			X	X
	Implementation climate—compatibility	Integration of the program support		X		X
	Readiness for implementation—leadership engagement	Leadership engagement			X	X
Characteristics of individuals	Knowledge and belief about the intervention	Personal experiences		X		
	Other personal attributes	Personality traits			X	
Process	Planning	Structure			X	
	Executing	Integration of screening activities			X

**Table 3:** Barriers to CRC screening activities in SFCCC by informants’ roles according to CFIR

CFIR domains	CFIR constructs and subconstructs	Themes	Roles			
			**SFCCC leadership**	**Medical directors**	**QI staff**	**Screening champions**
Outer setting	Needs and resources of patients	Housing instability	X	X	X	X
		Patients’ hesitancy to doing tests that involve stool			X	X
		Patients’ poor adherence to completing screening		X		X
		Unmet basic needs		X		X
Inner setting	Structural characteristics	Different screening programs in clinics	X			
	Implementation climate–relative priority	Other priorities in the clinics			X	X
	Readiness for implementation—leadership engagement	Lack of engagement from providers and leadership			X	
	Readiness for implementation—available resources	Lack of staff	X	X	X	X
		Staff turnover		X	X	
		Lack of materials			X	X
Process	Planning	Poor tracking of information		X	X	
		Lack of an organized strategy after patients have an abnormal FIT		X		X
	Executing	Failures on the implementation of screening strategies		X	X	

### Facilitators

Participants identified the factors that supported the implementation of CRC screening strategies, which corresponded to four CFIR domains: intervention characteristics, inner setting, characteristics of individuals, and process.

#### Intervention characteristics

We identified three constructs in informants’ answers related to this domain: *adaptability*, *design quality and packaging*, and *cost*. The most salient facilitator themes were technical assistance and stipends offered by the CRC Screening Program. Regarding technical assistance, informants across roles mentioned the approach used by members of the Task Force supporting the implementation of screening strategies.

A healthy level of engagement and a realistic level of engagement in light of competing priorities. QI director

Similarly, stipends were motivating, and respondents from all roles described how the monetary support was useful to their work.

The grant funds helped to implement screening activities. We were able to offset the extra responsibility given to the screening champion with a little extra incentive, which is always helpful for staff morale. Get folks to jump into the project gladly rather than reluctantly. Medical director

Informants also emphasized the constant communication from task force members, their awareness of the work conducted at the CHCs, and their flexible not punitive attitude facilitated the implementation of screening activities.

Great communication. Not being pushy with us and really understanding that we have limited capacity, we have limited staff, we have limited time and resources, and having another project on our plate can be overwhelming for us. QI manager

Other characteristics of technical assistance mentioned were accountability (medical directors, QI staff, and screening champions), expertise (SFCCC leaders and QI staff), and materials provided (medical directors, QI staff, and screening champions).

One of the most helpful things has been a feeling that there’s an outside source coming in that really keeps us alert and, on our toes. It’s just emotional and feeling of support is encouraging. Screening champion

#### Inner setting

Within the inner setting domain, the constructs identified were *networks and communications*, *implementation climate* (*goals and feedback* and *compatibility* subconstructs), and *readiness for implementation* (*leadership engagement* subconstruct). The QI staff and screening champions said that communication and collaboration within CHCs, having the opportunity of discussing their goals and strategies as a team, and leadership engagement were key elements that facilitated their work.

A lot of it [successful implementation of screening activities] stems from our communication and messaging to the team involved, but also our entire organization. QI director

Informants also highlighted that integrating support received from the CRC Screening Program into their daily work facilitated screening activities.

I think the whole idea with this program is that it helps us put in place a screening that becomes just part of the visit. And once those things get integrated, it becomes a normal part of the process, it becomes a habit, it just sticks. Medical director

#### Characteristics of individuals

Within the characteristics of individuals domain, the constructs identified were *knowledge and belief about the intervention* and *other personal attributes*, both demonstrating how personal experiences and personality traits of people working on the screening activities helped during the implementation of CRC screening strategies.

Just the motivation that the staff had to do it, the interest, there was some personal experiences of staff who have had family members with CRC. I think that hearing personal stories also helped kind of motivate the QI team. QI manager

#### Process

Within the process domain, p*lanning* and *executing* were described by the QI staff. Informants described how support received from the CRC Screening Program helped the clinic improve the structure of screening programs and integration of screening activities at different levels.

This project gave us a way to be a little bit more organized with [CRC screening] and a little bit more coordinated so that we could do better follow-up with our patients. QI director

### Barriers

Participants also identified factors that hindered the implementation of CRC screening strategies in CHCs, corresponding with three of the five CFIR domains: outer setting, inner setting, and process.

#### Outer setting

All themes identified as part of the outer setting related to patients’ characteristics that impeded them from participating in screening programs (*needs and resources of patients* construct). Housing instability was the most salient theme, described by informants from all roles.

A lot of our patients are homeless, so then not even having a location in which to collect their stools. Screening champion

Another salient theme mentioned by medical directors, QI staff, and screening champions was patients’ hesitancy to do tests that involve stool, a common observation in all CHCs as FIT is the main CRC screening strategy in SFCCC.

Because a FIT test is so gross to do, obviously not everyone feels comfortable touching their feces… if there’s a way of removing that stigma that exists with the grossness that goes with it. QI director

Patients’ low screening completion (for both FITs and colonoscopies) was another challenge expressed by the QI staff and screening champions.

Patients either do not want to do colonoscopies or further FITs that they would just not follow-up on completing and mailing them in. Despite multiple [QI initiatives] to do outreach around that, we never found a method that really connected with our patients. Screening champion

Medical directors and screening champions considered unmet basic needs (e.g., food, housing) as important limiting factors for patients to complete the screening tests once they were recommended by the CHC staff.

Even if they have a history of CRC in their family, because they’re so busy worrying about where they’re going to be and what they’re going to eat. It’s a struggle because you have all the social determinants working against them. And on top of it, we’re trying to do this other prevention as well, which may not seem as impactful for them. Medical director

#### Inner setting

We identified the barriers related to three constructs of the inner setting domain: *structural characteristics*, *implementation climate* (*relative priority* subconstruct), and *readiness for implementation* (*leadership engagement* and *available resources* subconstructs). As mentioned earlier, SFCCC includes many clinical sites and different screening programs, which SFCCC leaders described as challenging to support all the QI work conducted at each site.

They [CHCs] are at different stages of development, some are very advanced, some not so advanced. To offer a product that meets people where they’re at, or to offer technical assistance, it’s very difficult and challenging. SFCCC leader

The QI staff and screening champions also pointed out the existence of other priorities in the CHCs, unrelated to the pandemic, that interfered with CRC screening activities.

We’re not just focusing on CRC screening, there are other priorities as well. We have a lot of tasks that we want to do really well in. Screening champion

The QI staff raised concerns about the lack of engagement from providers and leadership of the CHCs with respect to CRC screening activities (pre-pandemic).

Provider buy-in, engaging the provider into the quality improvement program. Specifically, our FIT test. So that was a little bit of a challenge. QI director

Another barrier mentioned by SFCCC leadership, medical directors, QI staff, and screening champions, was understaffing for CRC screening-related activities.

When we’re understaffed, everyone’s working more, less effectively because we’re slammed and we would have times when we had no FIT tests put together. Screening champion

The medical directors and QI staff described that in addition to understaffing, the recurrent staff turnover limited the work conducted at the CHCs.

Sometimes you start something and then you have a lot of turnover of staff and then you have to kind of start all over again, getting people trained up and getting them involved and trained into the process as well as understanding what the work is that we’re doing. Medical director

Lack of materials (e.g., low inventory of FIT kits and language-appropriate educational materials) was another barrier identified by the QI staff and screening champions.

We also had problems with obtaining all of the materials we needed for having complete FIT tests. Screening champion

#### Process

Within the process domain, informants mentioned barriers related to two constructs: *planning* and *executing*. The medical directors and QI staff described how the poor tracking of clinical data was a limiting factor when CHCs started planning their CRC screening strategies.

When we first started, we had no idea what our screening rates were. Unfortunately, our EHR wasn’t designed in the best way to provide that kind of information, so we started from scratch. QI manager

Another common theme mentioned by medical directors and screening champions was the lack of an organized strategy after patients have an abnormal FIT. They identified problems with tracking information and reporting results.

I think the follow-up is something that we need to make sure we have a better tracking of, not just referral to our gastroenterologists, but actually getting that colonoscopy report back. Medical director

Also, medical directors and QI managers described the unsuccessful implementation of screening strategies (i.e., mailing FITs).

There was at some point a consideration in a discussion of how to use mailings and the cost and an analysis and examination of all that. That never happened. Medical director

### Impact of the COVID-19 pandemic

An important theme that emerged in all conversations was the impact of the pandemic on non-urgent patient care, even before direct questions regarding COVID-19 were asked. In our evaluation, however, we did not classify the pandemic under any of the CFIR domains because none of the existing constructs illustrates an event of this nature.

In Fig. 1, we show the CRC screening rates of CHCs participating in the study before and after the onset of the pandemic. CRC screening rates increased slightly in 2019 (pre-pandemic) and had a continuous decline in the first three quarters of 2020 (pandemic), followed by a sharp but moderate increase in the last quarter of 2020 that persisted in quarters of 2021. By the end of 2021, CRC screening rates in SFCCC reached pre-pandemic levels.

Informants from all roles had similar responses when talking about their experiences related to the pandemic:

That [pandemic] was the largest factor. I can’t really think of a factor that would have really prevented us from moving forward. That changed everything. QI director

Participants described how different aspects of the pandemic impeded the CRC screening work they were doing and stalled their progress (Table 4). The SFCCC leaders and QI staff alluded to the negative effect of local “shelter in place” orders on basic clinic functions, as patients’ visits to CHCs reduced drastically. The predominant sentiment in the early days of the pandemic, described by interviewees from all roles, was that CHCs’ priorities, resources, and focus shifted to ameliorate the impact of the new virus in the communities they serve, rather than on CRC screening. For example, the CHCs’ staff working on CRC screening were diverted to COVID-19-related activities (i.e., testing, vaccination). Also, the SFCCC leaders, QI staff, and screening champions mentioned that most of the patient care shifted to telemedicine, and they were not fully prepared for the change in a way that CRC screening could continue, as patient outreach and distribution of FIT kits usually occurs during in-person visits. Informants described that after a few weeks, CHCs resumed non-urgent activities, but they had a hard time determining the best approach that would allow them to serve patients while keeping social distancing measures. The SFCCC leaders, medical directors, QI staff, and screening champions manifested a sense of uncertainty around CRC screening strategy implementation.

**Table 4:** Informants’ quotes of themes related to COVID-19 and its impact on CRC screening activities

Themes	Quotes
*COVID-19*	
Pandemic	QI director: “That (pandemic) was I think the largest factor. I can’t really think of a factor that would have really prevented us from moving forward. That changed everything.” Screening champion: “Yeah, so we did great before the pandemic in increasing our colorectal cancer screening. And then after the pandemic, our numbers decreased precipitously.”
Shelter in place order	SFCCC leader: “Well, initially in the pandemic, in the shelter in place order in San Francisco was on, I think March the 15th or somewhere around there so starting in late March and April, initially many of the clinics greatly reduced in-person visits.” QI manager: “I guess probably the biggest challenge, and maybe it was just when the pandemic started last year, all our screening was put on hold for a little bit because people were more worried about, obviously like shelter-in-place and the pandemic and protecting themselves.”
Competing demands during crisis	QI manager: “All of the QI staff were now… They were in the COVID tents, they were doing testing. So not only were the patients not coming in for the test, but our staff were just not on QI, it basically almost completely halted. We stopped having QI meetings for a window of time.” Screening champion: “COVID has been a huge challenge. I think screenings, not just colorectal cancer screenings have been a challenge to remember, because you have to do extra screenings around COVID and then there’s only so many minutes you have with every patient and our patients are very complex. So I think remembering those routine screenings has definitely been a challenge.”
Shift to virtual visits	SFCCC leader: “The numbers dropped dramatically, both because of the drop in in-person visits and the newness of trying to get screening done when you were doing most of your visits virtually.” Screening champion: “Well, the main issue is that we have to somehow get a physical test kit to a patient. Usually that was done in per person in clinic. And now that we’re not having as many people come in and switching most of our services over to telehealth. That has just complicated things.”
Uncertainty with CRC screening strategies	SFCCC leader: “It’s just that both they (clinics) and us are still struggling to find the optimal way to do routine things like colorectal cancer screening in the middle of this pandemic.” Medical director: “So we’re impacted because we recognize that we are not doing as well on screening. And the primary challenge I think that we had, is that we rooted our screening on the anticipation of the personal interaction and education that came from patients who are coming to our clinic.”
CRC Screening Program support—maintaining the focus on CRC screening	SFCCC leader: “SF CAN has maintained the focus on colorectal cancer screening. What happens with the pandemic is that you lose that focus for all the reasons. The focus shifts to COVID and so most clinics are, for that matter, most large health systems and most providers have seen drops their regular QI measures during the pandemic. We have also made a pitch to our clinics to do something similar with breast cancer.”
CRC Screening Program support—availability	QI director: “I was, basically, taking on the program when we were entering the pandemic. But based on my experience overall in working with SF CAN, it’s been very positive. They’ve been very responsive with our needs and anything basically that we seek of their support.” Screening champion: “I think they have been really available and proactive in reaching out to us. They’ve been understanding of our challenges and really trying to keep up with how our clinic is handling the pandemic. I don’t think it has changed very much other than not having in-person meetings, but they’ve been just as available to us.”

Interviewees also described the support received from the CRC Screening Program after the onset of the pandemic. They highlighted the help received for maintaining the focus on CRC screening (SFCCC leader and medical director) and the availability of the task force members despite the absence of in-person meetings (QI staff and screening champion).

### Needs and recommendations

Respondents provided insight into their needs and recommendations for improving the support they receive from the CRC Screening Program (Table 5). Having additional staff and support for buying FIT kits and completing their mailings were the most mentioned needs. QI managers and screening champions described the importance of having additional staff dedicated to their CRC screening programs, as they see the potential for doing a better job in the area of patient follow-up (for both completion of FIT and colonoscopies). The QI staff and medical directors expressed their interest in incorporating mailed FITs into their strategies but needed support for obtaining high-quality FIT kits and preparing them to be sent. Interviewees also manifested that CHCs needed more educational materials for the staff and patients (QI staff), access to protocols used successfully in other CHCs (QI staff), trainings for new staff (screening champion), and advocates for QI work and their demands of additional resources (QI staff).

**Table 5:** Informants’ quotes of needs and recommendations for the CRC Task Force

Themes	Quotes
*Needs*	
Additional staff	QI manager: “Honestly, the first thing that comes to mind is more staffing just internally, just because people are so short on time with all of the duties that they already have and honestly, just a little bit more planning on our end. I feel like, because there’s a lot of potential that we can do. We just haven’t implemented mail FIT for example, or any of those things.” Screening champion: “Of course always having additional staff or correct amount of staffing just to make sure that patient care is being met.”
Support for FIT kits/mailed FIT kits	Medical director: “Getting the supports to do mailings because you couldn’t have a better rationale to do that than COVID, that would be the major. And we’d have to find a way so that the clinic was significantly supported in a way where there were the financial resources to say, ‘Let’s do this as a pilot, and get the data and learn during COVID, how effective can this be?’” QI director: “I think purchasing the colorectal cancer screening test kits, and providing them to us in addition to the funding would be ideal. This way, we’re using a FIT kit that they have vetted, or SF CAN has vetted and purchased and provided to us.”
Patients’ outreach and follow-up	
Educational materials	QI manager: “Maybe if there can be more educational resources, that would be helpful that we can disseminate and share with others in our clinic. And that would be for staff, like staff education as well as patients education I think.”
Other materials—protocols	QI manager: “I think if we were to restart working with SF CAN, what will be helpful is just some basic outline of a protocol that’s worked with another clinic to get started. Like a guide plan to getting involved in the screening efforts again.”
Trainings	Screening champion: “I think for right now that is maybe just another in-person or virtual teaching training session, like they did, it was probably over a year ago, to train any of our new staff that have come on since then.”
Support/advocacy QI efforts	QI manager: “I would say, helping to advocate for the QI staff, that they need more time to get results, because one hour a week is not enough, but the only way to change that is for the senior level, and executives to agree to make some big changes. And I think having extra support to advocate for that because that’s the next step. Does that mean hiring another staff member? Does that mean giving one shift a week, so you have four hours a week or is it one day a week to your QI staff? And really cementing that culture in there. So I think that at some point, if you have kind of those voices from an outside person working together with the QI, to really move that transition, I think would be helpful.”
*Recommendations*	
Increase stipend	SFCCC leader: “I mean, they could give out more money to be devoted towards colorectal cancer screening.” QI manager: “From an admin perspective, I think support has been great. The financial piece, I would say could be a little more.”
Sharing lessons learned	Medical director: “To learn about best practices from other clinics and what other people are doing and how they’re addressing this with the homeless population and what kind of results people are getting and things like that.” Screening champion: “I think whatever kind of advice, or tips, or any sort of resources that other community health centers or just other health centers have learned or have created with addressing CRC screening when a lot of patients are not coming into the clinic
Additional resources	SFCCC leader: “You know, if SF CAN had infinite resources and could fund a staff person whose job it would be to coordinate cancer screening in the clinics, that would be great. Then there would be a dedicated resource in general, SF CAN doesn’t have that level of resource, but if they did, that would be great. If we had a staff person who was dedicated to cancer screening, then it would be a lot easier to accomplish that. The staff person could work directly with the clinics in terms of improving cancer screening measures.”
Have a regular schedule for meetings	QI manager: “I would say maybe keeping somewhat of a regular schedule on check-ins. My understanding is that they were a little bit more sporadic, but if there was, for example, a calendar that said every first quarter of each month, Monday, we meet with SF CAN, then I think that’s something that’s on the calendar that is setting a meeting with an agenda I think would have been helpful.”

Increasing the stipend amount (SFCCC leaders and QI staff), sharing lessons learned from other CHCs (medical directors and screening champions), and providing additional resources (SFCCC leaders and screening champions) were the most common recommendations for the CRC Screening Program. Regarding additional resources, informants mentioned how helpful it could be if SF CAN had a staff person dedicated to cancer screening and coordinating this effort across CHCs. Informants also recommended the inclusion of materials that help them to continue improving screening rates in the context of the pandemic. Another recommendation offered was to have a more frequent schedule of meetings or check-ins (QI staff).

## Discussion

In this study, we evaluated the influence of support provided by the SF CAN’s CRC Screening Program through its task force and identified the facilitators and barriers to CRC screening activities in a consortium of CHCs before and after the onset of the COVID-19 pandemic. Using CFIR, we observed facilitators related to intervention characteristics, inner setting, characteristics of individuals and process domains, and barriers in the outer setting, inner setting, and process domains. We documented how the COVID-19 pandemic hampered non-urgent care in CHCs, and they had to modify the way preventive services were offered. Although new strategies were adopted (i.e., mailed FITs, telemedicine), all CHCs were not prepared for the changes. The support offered by the CRC Screening Program was well-received both at the level of the SFCCC leadership and within CHCs that decided to participate in ongoing activities, but interviewees reported areas for improvement, specifically, sharing successful experiences and providing additional resources.

We found facilitators to CRC screening strategies in all CFIR domains with the exception of the outer setting. Interviewees across the roles highlighted many elements of the technical assistance provided by the task force, and their friendly and not-punitive approach was the most appreciated characteristic. Other aspects valued by respondents were having an outside partner offering a system of accountability, including guidance from experts, funding, and materials. These findings add to prior research documenting how technical assistance aids to enhance cancer prevention and control programs in the adoption of evidence-based interventions [19, 20]. Leadership engagement was the most salient theme of the inner setting domain, followed by positive interpersonal dynamics within CHCs related to communication, collaboration, and discussion of goals. Implementation science studies focused on CRC screening have also described these facilitators [13, 15, 21, 22]. In the characteristics of individuals and process domains, the themes were not mentioned by participants from multiple roles, but similar to other studies, personal attributes and experiences of people implementing the screening activities, as well as the quality of their work and commitment, were considered important [14, 15, 21].

Informants mentioned barriers to the implementation of CRC screening at different levels, represented in three CFIR domains. Barriers to the outer setting were mostly patient-level factors (i.e., housing instability, patients’ hesitancy to do the tests, having other priorities) that impeded them from completing the screening tests. These findings are consistent with research reporting patients having other priorities in their daily lives and their reluctance to handle feces as barriers to CRC screening [14, 23]. Also, considering the socioeconomic profile of the population served at SFCCC, the critical housing situation in San Francisco, and the process to complete and return a FIT kit, it is not surprising that housing instability was the most salient deterrent of CRC screening identified in our study. On the other hand, and in line with previous research, the inner setting barriers described by participants were related to the lack of resources (i.e., staff and materials) and lack of support from individuals in leadership positions within CHCs [13–15, 21, 24]. Finally, process barriers corresponded to limitations in the planning and execution of screening strategies, particularly in the collection and flow of information, and in the management of patients with abnormal FITs. Other studies conducted in similar settings have also described these issues [21, 25].

Early after the onset of the pandemic, experts of leading medical and public health organizations in the USA and worldwide warned about the imminent impact of the COVID-19 pandemic (and the measures taken to limit the spread of the virus) on the continuum of cancer and cancer outcomes in the population [26–28]. Due to the lockdowns, the physical distancing recommendations, and the COVID-19-related assignments for CHCs staff, CRC screening at SFCCC was interrupted for several weeks. Many CHCs adopted telemedicine, and in some cases, it became for a time the only contact patients had with the health system for non-urgent care. Some interviewees mentioned that mailing FIT kits was their solution to the limitation put on in-person encounters to continue CRC screening. Although there were CHCs that implemented mailing FITs successfully, others were not prepared to adopt this alternative and CRC screening activities stopped. At the time this study took place, CHCs had opened, but they were seeing a lower volume of patients. Six months later, screening rates had returned to the highest pre-pandemic level. We believe the observed recovery in screening rates was related to a combination of resumption of in-person care, re-engagement with screening processes implemented in the years prior to the pandemic, and, in some cases, the successful implementation and continuation of mailed FIT activities. Despite the overall progress at SFCCC, some CHCs have not recovered completely. Interviewees indicated a need for additional CRC Screening Program support to assist with screening in the context of a continuing pandemic. In general, participants spoke positively about the support received from the task force after the onset of the pandemic and appreciated the help in keeping their focus on CRC screening even when activities had to be temporarily suspended.

CHC leadership engagement was a key theme during the interviews, considered a facilitator to CRC screening as well as a barrier if lacking. Indeed, not all CHCs chose to invite help from the CRC Screening Program, and frequent leadership turnover in some of the sites limited the ability of the task force to continue to engage successfully over time. Conversely, one CHC ended its partnership with the CRC Screening Program after 2 years because its strong leadership team no longer needed external support. Previous studies have found clinic leadership to be a crucial component for the implementation of CRC screening in safety net health systems [15, 21, 22]. Other studies have suggested that organizational readiness should be assessed prior to attempting interventions such as this. While we very much agree that such assessments are likely to improve implementation success, we also found that external pressures and internal staffing changes can rapidly alter a clinic’s motivation and capacity to successfully implement CRC screening [29]. By remaining available and flexible in its support for SFCCC and its member clinics over a period of several years, the CRC Screening Program has attempted to adjust to the changing terrain and make it easier for clinical teams to take advantage of training and resources at times when leadership and clinical teams are ready to move their screening efforts forward.

While the overall perception of the SF CAN partnerships with SFCCC and its CHCs has been extremely positive by the stakeholders interviewed, there is less clarity about the partnership’s impact on measurable outcomes such as screening rates and even less so with regard to longer-term outcomes such as CRC early diagnosis and treatment. With the data available, it is not possible to ascertain whether screening rates would have been maintained or recovered as well as they did after the pandemic without the task force support. Clearly, many of the activities supported by the CRC Screening Program were severely undermined by the pandemic, especially as the pandemic disproportionately affected populations served by CHCs [30–32]. Regardless, we have observed many screening processes that have improved as a result of this project. SFCCC now reports screening rates for the subset of patients who are homeless, and awareness of low screening rates in this group has led to new approaches targeting this population. The task force has identified an overnight facility accessible to SFCCC’s CHCs that can provide a safe place to complete a colonoscopy bowel prep with free transportation to and from colonoscopy when needed. All participating sites now use an evidence-based single-sample FIT for initial screening, supported by wordless instructions, and some sites have begun using small patient incentives to encourage higher rates of FIT return. All sites now actively track abnormal FIT and completion of colonoscopy thereafter. As mentioned above, some sites have introduced mail and telephone outreach strategies to offer screening and follow-up to those who are not regularly seen in person. The frequent contact and prioritization of CRC screening over time have contributed to the perception that screening is important and possible, even in low-resource settings serving patients with multiple competing medical and social needs [33].

The need for training and technical assistance with regard to specific evidence-based interventions for CRC screening at the clinic level has been well-documented and addressed by the US Centers for Disease Control and Prevention through its CRC Control Program [34] and the National Cancer Institute (NCI) through its Screen to Save Program [35]. NCI-designated Cancer Centers each have a self-defined geographic area that it pledges to serve through community engagement and outreach [36]. SFCAN is one model for community engagement and outreach to address the cancer screening needs of diverse communities served by CHCs that is potentially sustainable and scalable with institutional resources from well-resourced NCI-designated cancer centers, though of course even these resources are limited. Further research should explore alternative approaches to engage CHCs and provide ongoing access to training and technical assistance within their catchment areas to maximize benefits achieved relative to available resources.

This study had limitations. First, while purposive sampling helps to increase the representation of specific characteristics in the sample, it is not a representative sample and may not fully represent all stakeholders engaged by the CRC Screening Program and its task force. Second, although researchers external to SF CAN conducted the interviews, study participants might have felt compelled to describe positive attributes and were reluctant to critique their work. Third, in this study, we decided to focus on the perspectives of individuals working on CRC screening at SFCCC and did not include patients. Some of the barriers identified by respondents were centered on patients, pointing to the potential value of confirming our findings in a group of SFCCC patients. Fourth, we conducted this study in an urban setting, and our results might not be generalizable to other contexts, especially CHCs in rural areas.

## Conclusions

This study provides insight into the perceived impact of SF CAN and its CRC task force on CRC screening processes within the SFCCC and its member CHCs, both before and during the COVID-19 pandemic. The use of CFIR allowed for the identification of factors that affect CRC screening activities at multiple levels and the extent to which the CRC Screening Program was able to help SFCCC address them. The timing of the evaluation allowed for novel insights into the feasibility and sustainability of such partnerships during a public health emergency such as the COVID-19 pandemic. Our SF CAN model of partnership with SFCCC is a promising example of collaboration that deserves further examination and replication by others that wish to prioritize the goal of greater access to high-quality CRC screening in every community.

## Supplementary Information

**Additional file 1.** Interview guide.

**Additional file 2.** Standards for Reporting Qualitative Research (SRQR).

**Additional file 3:** **Table S1.** Informants’ quotes of facilitators to CRC screening activities in SFCCC according to CFIR. **Table S2.** Informants’ quotes of barriers to CRC screening activities in SFCCC according to CFIR.

## Data Availability

The dataset used and analyzed in this study will not be shared to protect participants’ privacy and confidentiality.

## Abbreviations

CFIR: Consolidated Framework for Implementation Research
CHCs: Community health centers
CRC: Colorectal cancer
FIT: Fecal immunochemical test
NCI: National Cancer Institute
QI: Quality improvement
SF CAN: San Francisco Cancer Initiative
SFCCC: San Francisco Community Clinic Consortium
UCSF: University of California San Francisco
