# Supplementary material for: Implementation of a novel program to support colorectal cancer screening in a community health center consortium before and after the onset of COVID-19: a qualitative study of stakeholders’ perspectives
Source: Implement Sci Commun. 2023 May 22;4:54. doi: 10.1186/s43058-023-00439-x (PMC10201507; doi:10.1186/s43058-023-00439-x)
Supplement: Supplementary file 1 — Additional file 1. Interview guide. [file 43058_2023_439_MOESM1_ESM.docx]

**Interview guide-** SFCCC leadership

**Role and goals**

1. What are your roles at SFCCC and how long have you been in this position?
2. What factors influenced your decision to work with the SF CAN’s CRC Screening program?
3. What were/are the goals in participating of the CRC screening program?
4. Can you please tell me about how is it going in achieving these goals?

*If goals have been achieved*, what factors helped to reach the goals?

*If goals have not been achieved*, what factors prevented from reaching the goals?

**Support and communication**

1. What kind of support do/did you receive from the SF CAN’s CRC Screening program?
2. What kind of communication do you have with members of the SF CAN’s CRC Screening program?
3. How often do you communicate (meet or talk)?
4. What kind of communication do you have with clinics’ medical directors or QI managers?
5. How often do you communicate (meet or talk)?

**COVID-19**

1. How has the COVID-19 pandemic changed the services provided at the clinics?
2. For how long services have been/were interrupted?
3. How did screening activities change after the onset of the COVID-19 pandemic?
4. What do you think will be needed to:

resume activities?

reach pre COVID-19 participation?

1. What do you think of the support received from the program (before and after the onset of the pandemic)?
   1. What has been helpful?
   2. Is there anything you think should be done in a different way?

**Future**

1. What type of support do you think would be useful for implementing/continuing screening activities in the future?
2. After the program ends, what are the plans for continuing CRC screening activities?
3. What other resources might be needed?
4. Is there anything else you would like to share?

**Interview guide-** Medical director/QI staff/Screening champion

**Role and goals**

1. What is your role at ­­­­­_____ and how long have you been in this position?
2. What were/are the goals in participating of the CRC screening program?
3. Can you please tell me about how did it go in achieving these goals?

*If goals have been achieved*, what factors helped to reach the goals?

*If goals have not been achieved*, what factors prevented from reaching the goals?

**Support and communication**

1. What kind of support do/did you receive from the SF CAN’s CRC Screening program?
2. What kind of communication do you have with members of the SF CAN’s CRC Screening program?
3. How often do you communicate (meet or talk)?
4. What kind of communication do you have with staff in charge of implementing screening activities at the clinic?
5. How often do you communicate (meet or talk)?

**Planning and implementation**

1. Can you describe in detail what screening activities you are/were doing?
2. How did you decide to implement these strategies?
3. What factors influenced the decision?
4. Who was involved in the decision?
5. Can you talk about how the screening activities are/were implemented?
6. What staff are/were involved in implementation?
7. What are/were their responsibilities?
8. Were staff hired for implementing screening activities?
9. What factors helped to implement the screening activities?
10. What are/were some of the challenges in implementing the screening activities?
11. What actions are/were taken to overcome the challenges?
12. How do you think screening activities are/were seen by other members of the clinic?
13. Are/were members of the community served by the clinic involved in planning or implementation of screening activities?

*If yes*, how are they contributing?

1. Were you doing any of these screening activities before receiving support from the program?

*If yes*, for how long?

1. Can you describe screening activities before receiving support from the program?

**COVID-19**

1. How has the COVID-19 pandemic changed the services provided at the clinic?
2. For how long services have been/were interrupted?
3. What screening activities/strategies have resumed?
4. What screening activities/strategies have still not resumed?
5. How did screening activities change after the onset of the COVID-19 pandemic?
6. What do you think will be needed to:

*(if not resumed)* resume activities?

*(if resumed)* reach pre COVID-19 participation?

1. What do you think of the support received from the program (before and after the onset of the pandemic)?
   1. What has been helpful?
   2. Is there anything you think should be done in a different way?

**Future**

1. What type of support do you think would be useful for implementing/continuing screening activities in the future?
2. After the program ends, what are the plans for continuing CRC screening activities?
3. What other resources might be needed?
4. Is there anything else you would like to share?
