# Supplementary material for: Implementation of a novel program to support colorectal cancer screening in a community health center consortium before and after the onset of COVID-19: a qualitative study of stakeholders’ perspectives
Source: Implement Sci Commun. 2023 May 22;4:54. doi: 10.1186/s43058-023-00439-x (PMC10201507; doi:10.1186/s43058-023-00439-x)
Supplement: Supplementary file 3 — Additional file 3: Table S1. Informants’ quotes of facilitators to CRC screening activities in SFCCC according to CFIR. Table S2. Informants’ quotes of barriers to CRC screening activities in SFCCC according to CFIR. [file 43058_2023_439_MOESM3_ESM.docx]

| **Supplementary Table 1. Informants’ quotes of facilitators to CRC screening activities in SFCCC according to CFIR** | | | |
| --- | --- | --- | --- |
| **CFIR domains** | **CFIR constructs and subconstructs** | **Themes** | **Quotes** |
| Intervention characteristics | Adaptability | Approach | SFCCC leader: “They try to do as much of the administrative work as possible for both us and the clinics. Recognizing that our strength is not the administrative aspect of a PDSA or quality improvement cycle. They've recognized that. So when I've been involved with them at the small group meetings with each individual clinics, they make it clear that they just want the clinics to come and be ready to share their experience and whatever they need for, and they don't put that burden on either us or the clinics to do.”  QI director: “It was a healthy level of engagement and a realistic level of engagement in light of competing priorities. I remember that was pre pandemic. I think the onus was really on us, on me and on the medical director and director of operations to move this forward. I felt as if SFCAN was doing their part to engage us and to provide resources. They were very available. They were always trying to schedule meetings with us.”  QI manager: “Great communication. Not being pushy with us and really understanding that we have limited capacity, we have limited staff, we have limited time and resources, and having another project on our plate can be overwhelming for us. But really working at our pace, checking in, but not being authoritative, not being like, "You were supposed to give us this report right now!" Which would've made me not want to work with them. If they're making me more stressed in my job.” |
|  | Design quality and packaging | Accountability | Medical director: “I think mostly just the focus and the timeline of accountability. So, that was actually much more helpful than specific training interventions. Ultimately what really mattered the most was that just knowing that for accepting some funds, we were holding ourselves accountable to that improvement. So it pushed us to achieve that.”  QI director: “And I think the relationship with SFCAN, it's a monthly reminder to us to look in and see how we're doing as a team with colorectal cancer screening.”  QI manager: “I think it's important to keep focus on measures and keep ... With focus, I mean occasional reminders. So, regular check-ins with SF CAN staff, I think was useful. I think it's important to remind us that this thing is going on and not to forget. The feedback that I got from staff that are working on this project was very high.”  Screening champion: “Honestly, I think one of the most helpful things really has been a feeling that there's an outside source coming in that really keeps us alert and on our toes. It sort of gives us a feeling that we're answerable to another entity and that we are partnering with another entity. It's just emotional and feeling of support is encouraging.” |
|  |  | Expertise | SFCCC leader: “They recognize our model that each individual clinic has their own QI efforts and they attempt to meet each individual clinic where they're at, and they have the technical expertise in this particular matter, in a way that we don't.”  QI manager: “So a lot of it's just that expertise and being able to talk through possible options, even if, sometimes we weren't able to.” |
|  |  | Materials | Medical director: “I really did like receiving the PDSA cycle template that they use because it was better than the one that I was using. So that was a nice little improvement.”  QI director: “I think the wordless instructions is really helpful for our patients, especially for those that English is not their first language. It's a way to give clear instructions and improve the percentage of people that actually go home and complete the FIT test. The wordless instructions has been really helpful.”  Screening champion: “SF CAN provided us with birthday cards. So we sent out birthday cards to patients when they turned 50. SF CAN also provided us with instructions, written instructions, to include with the colorectal cancer screening kits, the FIT tests.” |
|  |  | Trainings | Screening champion: “I honestly think that the in person training was the most helpful, because it gave us the language around screening to talk to patients about some or about a topic that could otherwise be a little bit awkward for some of our staff to talk about with our patients. I think that has the biggest beneficial effect on our screenings.” |
|  | Cost | Stipends | SFCCC leader: “The small grants that SFCAN gives to the health centers have helped them to focus on this measure (CRC screening rate).”  Medical director: “The grant funds helped to implement screening activities. We were able to offset the extra responsibility given to the screening champion with a little extra incentive, which is always helpful for staff morale. Get folks to jump into the project gladly rather than reluctantly.”  QI manager: “The financial support that came with participating in this grant was certainly a big factor that helped us in purchasing the FIT kits. Without that financial support, without that piece, I think it would be much harder.”  Screening champion: “Well, obviously the financial support helps us actually carry out all of these screenings.” |
| Inner Setting | Networks and communications | Communication | QI director: “I think a lot of it (successful implementation of screening activities and increase in screening rates) stems from our communication and messaging to the team involved, but also our entire organization. So I think I might've mentioned that CRC screening is one of our kind of top ... it's one of our screening measures that we put a lot of emphasis on. And so I think the messaging to our team and yeah ... I would say that a lot of it just has to do with people involved, people directly involved and also staff who may not be directly involved. They're kind of all aware that CRC screening is very important to our organization.” |
|  |  | Collaboration within clinics | Screening champion: “I think definitely inter departmental collaboration. I think kind of the main component that has been really helpful is just trying to get everybody on the same page, understanding the reasons why.” |
|  | Implementation climate- goals and feedback | Discussing goals and strategies as a team | QI manager: “I think having dedicated time to discuss that specific topic, because we had maybe half an hour or an hour monthly meeting to discuss what was going on, what our outreach efforts were and what our goals were. We also did a little bit of time in that, in those meeting sessions to do trainings on general, like PDSAs cycles and things like that, which helped inform us on how to do our strategies and attempts.” |
|  | Implementation climate- compatibility | Integration of the program support | Medical director: “I think the whole idea with this program is that it helps us put in place a screening that becomes just part of the visit, right? The way we screen for tobacco now, and when everybody started doing tobacco cessation 10 years ago it was something that was new, but now it's a habit, right? So I think it's the same thing with these kinds of prevention screenings. Did it get integrated into the EHR so that it's part of the MA screening when they're prepping the visit? Things like that. And once those things get integrated in and it becomes kind of a normal part of the process, it becomes a habit, it just sticks.”  Screening champion: “Yeah, so we kind of just through the objectives of the grant, we implemented this report that would get sent out to our providers and our clinical staff on kind of the patients who screened positive, but have not completed their colonoscopy yet. And that was meant to just help kind of consolidate all of the information into kind of one file for our clinical teams. And that was really helpful. It worked. I think it's a continuing process right now. So in terms of just how this grant has helped us, it's helped us kind of dive deeper into asking more questions and identifying areas in which we want to focus more on in order to work towards the goal of closing the CRC screening loop.” |
|  | Readiness for implementation- leadership engagement | Leadership engagement | Screening champion: “Having someone who is ... like we have one of our medical directors speak to the providers, just letting them know what changes we're going to be making and kind of giving everybody that heads up beforehand.” |
| Characteristics of individuals | Knowledge and belief about the intervention | Personal experiences | QI manager: “Just the motivation that the staff had to do it, the interest, there was some personal experiences of staff who have had family members have colorectal cancer. So I think that hearing personal stories also helped kind of motivate the QI team.” |
|  | Other personal attributes | Personality traits | Medical director: “The primary factor is we had a quality improvement team that was excellent, and it included a particular employee that was very good in terms of the data. And she also helped to build the comradery and teamwork and did a lot of the communication that's needed to implement a project like that. So this only works if... It takes time and commitment. And primary care is so challenged by all of the many demands that it requires a focus committed effort. And I think that we had that.” |
| Process | Planning | Structure | QI director: “So this project gave us a way to be a little bit more organized with it (colorectal cancer screening) and a little bit more coordinated so that, that way, we could do better follow-up with our patients.” |
|  | Executing | Integration of screening activities | QI manager: “I would say resuming birthday cards was one of those factors, and having the ability of providing taxi vouchers to our patients as well so they can come to the clinic and pick up a FIT test. Also, getting our lab department involved so that if a patient has a lab appointment and they're doing their routine labs there's a standing order there now for CRC screening if they're due so they could complete that on the spot. The resource center, we also have the ability to do Poop on Demand. So for patients that we were doing a CRC FIT test, we would ask them if they had the ability to complete the test the same day and a lot of them were actually doing that. So we were seeing some results there as well.” |

| **Supplementary Table 2. Informants’ quotes of barriers to CRC screening activities in SFCCC according to CFIR** | | | |
| --- | --- | --- | --- |
| **CFIR domains** | **CFIR constructs and subconstructs** | **Themes** | **Quotes** |
| Outer Setting | Needs and resources of patients | Housing instability | QI manager: “I think our number one barrier are the living conditions of our patient population. We're a community clinic located in [specific area] and a lot of our patients are unhoused, homeless.”  Screening champion: “A lot of our patients are homeless, so then not even having a location in which to collect their stools.” |
|  |  | Patients’ hesitancy to doing tests that involve stool | QI director: “Because a FIT test is so gross to do, I mean, obviously not everyone feels comfortable touching their feces… if there's a way of removing that stigma that exists with the grossness that goes with it.”  Medical director: “I would say that we have a pretty significant barrier in our patients' willingness to participate in the program, and just because of the hesitancy as it relates to stool and the kind of the education that's required around this.” |
|  |  | Patients’ poor adherence to completing screening | QI director: “So we had some struggles along the way, patient adherence being one of those through patient responsiveness, in returning the kits was one of the biggest challenges.”  Screening champion: “And we did get a number of pamphlets and materials to help work with our patients, but I think we still had a lot of issues with... patients would either not want to do colonoscopies or further FITs that they would just not follow up on completing and mailing them in. And despite multiple [QI initiatives] to do outreach around that, we never found a method that really connected with our patients.” |
|  |  | Unmet basic needs | Medical director: “Even if they have a history of colon cancer in their family, because they're so busy worrying about where they're going to be and what they're going to eat and things like that. So it's a struggle because you have all the social determinants working against them. And on top of it, we're trying to do this other prevention as well, which may not seem as impactful for them.”  Screening champion: “They have clearly other priorities that a lot of time more pressing than doing screening tests. So I think that's more challenging for our patient population in general, difficulty with having good rest, having adequate food, having adequate shelter. I think there's oftentimes it affects how our patients feel about doing these screening tests.” |
| Inner Setting | Structural characteristics | Different screening programs in clinics | SFCCC leader: “We have a lot of challenges because we're not a clinic ourselves, we're a consortium and the clinics have their own QI programs, or QI efforts. They're at different stages of development, some are very advanced, some not so advanced to be polite. So, to offer a product that meets people where they're at, or to offer technical assistance, to meet people where they're at is very difficult and challenging.” |
|  | Implementation climate- relative priority | Other priorities in the clinics | QI director: “Part of the year we do our federal reporting and our state reporting. And there's three to four months in which I am not thinking about anything else, but trying to do our reporting. And so then I don't have the motivation or the ability to push the other staff, who then don't have the time anyways, to move things forward. So I think that is, and those competing priorities of when all of a sudden you have a new focus that you have to work on or a new grant that kind of takes that time away.”  QI manager: “Maybe it's just because we're a busy clinic, sometimes there's competing priorities as far as when you're in clinic, that you're busy working with a provider or something else comes up or there's another project that comes up. Sometimes prioritizing, things fall to a lower priority sometimes when there's something else going on in the clinic.”  Screening champion: “I would say that challenges, I think, for a community health center, for our team at least, we're not just focusing on CRC screening. It's like there are other priorities as well. So I think sometimes it's just, we have a lot of tasks that we want to do really well in.” |
|  | Readiness for implementation- leadership engagement | Lack of engagement from providers and leadership | QI director: “Provider buy-in, engaging the provider into the quality improvement program. Specifically our FIT test. So that was a little bit of a challenge.”  QI manager: “I know SF CAN had some grant opportunities for us to do some continued work in that area and our leadership chose not to move forward with those grants. So we ended up basically just ending the program at that point. So maybe lack of internal support for it.” |
|  | Readiness for implementation-available resources | Lack of staff | SFCCC leader: “The biggest thing is always just resources. Like if you could get me enough, another staff person and just free up one of our staff person to have time to do it up to this. SFCAN is not the kind of organization that comes in and says, ‘Hey, here's another [staff person] or half-time [staff person].’ I mean, that's always feel what's needed.”  QI manager: “We had some staffing shortages and so we couldn't put as much resources into the program as we might have wanted to.”  Screening champion: “Another barrier was lack of staffing. So when we're understaffed, everyone's working more, less effectively because we're slammed and we would have times when we had no fit tests put together.” |
|  |  | Staff turnover | Medical director: “Sometimes you start something and then you have a lot of turnover of staff and then you have to kind of start all over again, getting people trained up and getting them involved and trained into the process as well as understanding what the work is that we're doing.”  QI manager: “I think the main thing that I would highlight as a challenge when almost all the people who were in the initial group had left and not being replaced.” |
|  |  | Lack of materials | QI director: “Not having the material in Tagalog, which is one of the languages, or any of our Asian dialects. So that's been a barrier. We don't have the tools in those languages. So having someone of a native language explain to them, the importance of a FIT test, but not walking away with any literature, it's been that barrier.”  Screening champion: “We also had problems with obtaining all of the materials we needed for having complete FIT tests. So we would get the tubes and the instructions, but then we wouldn't have the material that the patient's put on the toilet to collect the fecal samples. So then we're like telling patients, "Oh, use aluminum foil or use a newspaper." It was very sloppy. So we... That was a real barrier for us. I would say that was the main barrier was materials and organizing and ordering materials. The place that we ordered materials from did not package everything together, which makes the most sense. So he would have to call different people to get the different materials, to put the kits together.” |
| Process | Planning | Poor tracking of information | Medical director: “When we started looking at our CRC goals, screening rate was pretty low, within the 50%. There were a lot of issues with that, either we're not capturing the data or we're just not doing very well.”  QI manager: “Well, when we first started, this was early, when we first started, we had no idea what our screening rates were. Unfortunately, our EHR wasn't designed in the best way to provide that kind of information, so we started from scratch, and then had to do a lot of digging into people's charts and then updating areas of their charts called flow sheets to even see where they were, if they had been screened, if they had had colonoscopy.” |
|  |  | Lack of an organized strategy after patients have an abnormal FIT | Medical director: “I think we're pretty good in screening, but I think the follow up is that something that we need to make sure we have a better tracking of the not just referral to our gastroenterologists, but actually getting that colonoscopy report back. So we want to make sure that whole process. We're getting better screening, but we need to get better with the follow up.”  Screening champion: “I think kind of just on a grander level, we realized that the follow up process is pretty difficult just based on some things such as getting the referrals. It's more about just like kind of getting patients to complete their colonoscopies, which we realized was probably one of our biggest challenges. We're trying to get patients scheduled for their colonoscopies, at least for our health center, the colonoscopies were done out-house. Like they're not done in-house. They're kind of done elsewhere. And I think when we add that component of not having like direct control over colonoscopy, that does add a bit of a challenge for us. So that's kind of what I would say was the biggest challenge from how I saw it at least.” |
|  | Executing | Failures on the implementation of screening strategies | Medical director: “There was at some point a consideration in a discussion of how to use mailings and the cost and an analysis and examination of all that. That never happened.”  QI manager: “I think one big factor would have been trying to implement a mailed FIT idea. We have not been fortunate enough to find either a vendor or a price point that's good for us. So we actually ask patients to come into the clinic to pick up a FIT test and then they have to come back to drop it off. So that's been one of those factors that has prevented us from patients not returning I guess their FIT tests. There's about a 60% unreturned rate.” |
